# The potential association between degree of mammographic spiculation and prognosis

**DOI:** 10.1186/s13244-025-02194-0

**Published:** 2026-02-02

**Authors:** Li Sturesdotter, Hanna Sartor, Hedvig Kristensson, Oskar Hagberg, Kristina Lång

**Affiliations:** 1 Diagnostic Radiology, Translational Medicine, Lund University, Lund, Sweden; 2 Unilabs Breast Unit, Skåne University Hospital, Malmö, Sweden

**Keywords:** Mammography, Breast cancer, Breast density, Prognosis, Survival

## Abstract

**Objectives:**

Mammographically spiculated breast cancer is frequently less aggressive than cancers with alternative appearances. This study aims to investigate whether the degree of spiculations relative to the tumor mass on mammography, termed the spic mass ratio (SMR), is associated with breast cancer characteristics and survival.

**Materials and methods:**

This retrospective exploratory single-center study analyzed mammograms from 161 women with spiculated breast cancer in the Malmö Diet and Cancer Study cohort (2004–2014). Radiologists segmented the tumor mass and the spiculation areas. The SMR was calculated by dividing the combined tumor and spiculation area by the tumor area alone. The subjects were stratified into tertiles with low, medium, and high SMR. The study examined associations between SMR and breast density, mode of detection, age, tumor size, estrogen receptor status, progesterone receptor status, human epidermal growth factor receptor 2 status, Ki67, histological grade, axillary lymph node involvement (ALNI), histological type, and breast cancer-specific survival, utilizing the Chi-squared test, ANOVA, Fisher’s exact test, Kaplan–Meier curves, and Cox regression.

**Results:**

The mean age was 68 years (range 55–91). SMR was statistically significantly associated with both age and breast density. No other significant associations were observed. Among the nine women with the highest SMR values, axillary lymph node negativity, estrogen positivity, and an overall low Ki67 index were noted.

**Conclusions:**

The SMR, representing the degree of spiculations relative to tumor mass, was not significantly associated with breast cancer survival or ALNI. Further research is necessary to explore the prognostic implications of extensive spiculations in spiculated breast cancer.

**Critical relevance statement:**

The degree of spiculation relative to the tumor mass is an unexplored mammographic feature that can be measured subjectively, as in this study. Extensive spiculation was associated with higher age and lower breast density. No certain conclusions could be drawn regarding the impact on breast cancer survival.

**Key Points:**

- The degree of spiculation relative to the tumor mass on mammography is an unexplored mammographic feature.
- A high ratio of spiculations in relation to tumor mass was associated with higher age and lower breast density.
- The nine women with a very high spiculation to tumor mass ratio were all axillary lymph node negative.

**Graphical Abstract:**

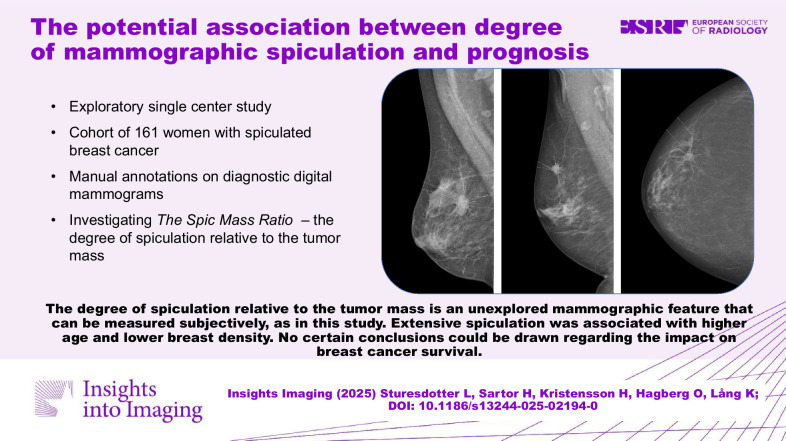

## Introduction

Breast lesions exhibit various mammographic appearances, including masses with spiculated margins [1]. Spiculation is a strong indicator of malignancy [2] and is commonly observed in low-grade tumors [3]. Typically, it suggests favorable tumor characteristics, such as estrogen receptor (ER) positivity and low histological grade [4–6]. However, aggressive tumors may also present with spicules, rendering spiculation a clinically inconclusive sign [2]. Spicules are radiating lines or strands extending from the central mass into the surrounding breast tissue on a mammogram, giving the tumor a stellate appearance. Histologically, these spicules are often associated with a desmoplastic peritumoral reaction characterized by reactive fibrosis [7]. It is plausible that the degree and configuration of spiculation observed in mammography reflect biologically significant tumor traits, though the exact nature and optimal measurement of this feature remain uncertain. We hypothesize that more extensive spiculation may reflect a beneficial immune response with potential prognostic significance.

Breast density is a significant factor to consider when studying spiculation, as it relates to the microenvironment composition in breast tissue and affects how tumors appear on mammography [8]. Tumors in women with dense breasts more frequently present as ill-defined masses or calcifications, while spiculation is less common compared to its occurrence in fat-involuted or moderately dense breasts [9]. The reason for this may be due to an intrinsic feature of the tumor biology or could be related to the lesser visibility of the spicules in dense tissue. The mode of tumor detection is another crucial aspect; clinically detected tumors generally have a worse prognosis than those detected through screening [10].

This study aims to investigate whether the degree of spiculations relative to the tumor mass on mammography, termed the spic mass ratio (SMR), is associated with breast cancer characteristics and survival. Additionally, it seeks to determine if this measure of spiculation can effectively stratify women with spiculated breast cancer into high-risk and low-risk groups.

## Materials and methods

This study received approval from the Ethics Review Board in Lund, Sweden (Official Records Nos. 652/2005, 166/2007, and 2014/830) and from the Swedish Ethical Review Authority (2022–04473–02). Conducted following the Declaration of Helsinki, it includes a subset of women from the Malmö Diet and Cancer Study (MDCS) cohort [11, 12]. Informed consent was obtained from participants during the baseline examination.

### Study population

**Fig. 1:**
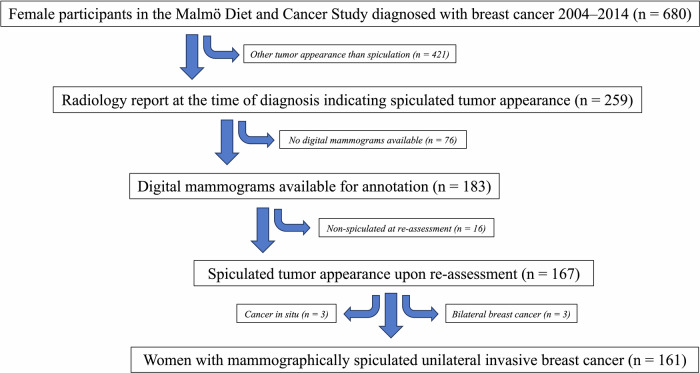
Flowchart illustrating the selection process of the study cohort

Data were collected from the female population in the prospective, population-based cohort MDCS (*n* = 17,035), initiated in 1991. The inclusion criteria required a breast cancer diagnosis from 2004 until the end of 2014, ensuring the availability of digital mammograms. All women diagnosed with breast cancer during this period were included consecutively. Additionally, the original radiology report was required to indicate a spiculated appearance. Exclusion criteria included non-spiculated appearance upon re-assessment of mammograms, cancer in situ, and bilateral breast cancer. Figure 1 presents a flowchart of the cohort.

### Mammographic information

The mammography reports of women with breast cancer in the MDCS have previously been reviewed with regard to dominant tumor appearance, breast density, and detection mode (screening vs. clinical), as detailed in prior studies [4, 9, 13]. Tumor appearance was categorized into several types, including the spiculated mass.

Breast density was qualitatively estimated based on the original radiology report from the diagnostic mammogram. The assessment included both breasts and all available views and was performed by one of five experienced breast radiologists (all with over 10 years of experience) at the Department of Breast Radiology. Breast density was classified into three categories based on the clinical routine at the Department of Breast Radiology in Malmö: fat-involuted, moderately dense, and dense breast parenchyma [9]. Fat-involuted corresponds to Breast Imaging Reporting and Data System (BI-RADS) 4^th^ edition category 1 (almost entirely fatty), moderately dense aligns with BI-RADS categories 2 and 3 (scattered fibroglandular densities and heterogeneously dense), and dense matches BI-RADS category 4 (extremely dense) [14]. As the data spanned the years from 2004–2014, the BI-RADS 4^th^ edition was applied for density categorization. Clinically detected cancers also include interval cancers.

### SMR

**Fig. 2:**
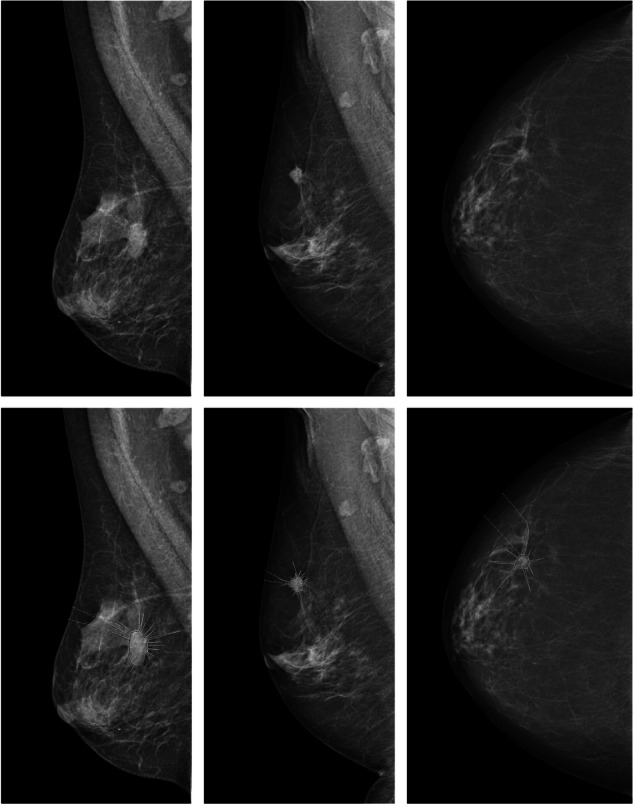
Examples of mammograms with spiculated tumors on the top row, and the same mammograms with corresponding SMR area annotations as they appear in the PACS system on the bottom row. Please note that the outlining of the tumor core and the spiculations was made subjectively by the reader(s), and that the core-spicules boundary was not operationalized with hard criteria. Left: mediolateral oblique view of the right breast, moderately dense parenchyma, and a spiculated tumor in the inner aspect of the breast. Middle: mediolateral oblique view of the right breast, moderately dense parenchyma, and a spiculated tumor in the cranial aspect of the breast. Right: craniocaudal view of the right breast, fat involved parenchyma, and a spiculated tumor in the lateral side of the breast

We developed the SMR parameter to facilitate the measurement of spiculation on mammograms. SMR is defined as the ratio of the tumor mass area, including spiculations, to the tumor mass area without spicules. Consequently, a greater degree of spiculation results in a higher SMR. The spiculated tumors were evaluated and annotated on mammograms by LS, at the time a resident in radiology with specialized training in breast radiology. In complex cases, measurements were conducted in consensus with an experienced breast radiologist, KL. The tumors were evaluated regarding the margin of the tumor mass. If there were spiculations visible to the naked eye, we considered the tumor spiculated and made the annotations on the view where they were most clearly visible. All mammograms were assessed on screens intended for radiologic use. The measurements were made based on the subjective tumor interpretation by the reader(s). No explicit criteria for the definition of the tumor core and/or the spiculations were employed. We utilized Sectra’s PACS software (Sectra AB, Linköping, Sweden) to annotate the mammograms. More specifically, the area measurement tool is used, where you click on several points along the border of the region of interest to obtain an area value. Firstly, the tumor mass area was measured, and after that (in the same session) tumor mass plus spiculation area was measured. Although we did not apply a strict criterion to define the mass border and the spicules, in most cases the margin was visible, with the radiating spiculations perpendicularly oriented to the mass border. Figure 2 illustrates three examples of SMR annotations.

To assess the stability of tumor size measurements, two independent readers evaluated the mammograms: a medical student (HK) measured the largest tumor diameter in millimeters, and LS measured the tumor area in square millimeters. The correlation between these two metrics was high (*r* = 0.87 (95% CI = 0.82–0.90), *p* < 0.001, Pearson’s correlation), indicating consistent and reproducible assessment across methods.

### Clinical information

Comprehensive clinical data on the women in the MDCS and their breast cancer diagnoses were sourced from the MDCS database. These data included vital status and causes of death, updated until December 31, 2018, via regular updates from the Swedish Cancer Register and the Swedish Cause of Death Register [9]. The median follow-up time for all included women (*n* = 161) was 8.45 years (range: 0.04–14.75; IQR: 5.63–10.76). For women who did not die from breast cancer (*n* = 143), it was 8.75 years (range: 2.60–14.75). The methods for data collection from medical records, radiology and pathology reports, and tissue microarrays in the MDCS have been described in detail previously [4, 9, 15–17].

In this study, the following variables were analyzed: age at diagnosis (continuous variable in years), tumor size (continuous variable in mm, based on pathology reports), ER and progesterone receptor (PR) status (binary: positive or negative), human epidermal growth factor receptor 2 (HER2) status (binary: positive or negative), Ki67 expression (ordinal: low, intermediate, high), histological grade (ordinal: 1, 2, and 3) according to Elston and Ellis [18], number of axillary lymph nodes with metastases (continuous), axillary lymph node involvement (ALNI) (binary: positive or negative), histological type (categorical with five groups and binary: ductal vs. non-ductal cancer), vital status (binary: alive or dead/emigrated), and cause of death (binary: due to breast cancer or another cause). No distinct cutoff points for Ki67 categories were established. Instead, values were divided into three equal groups (“low”, “intermediate”, and “high”) over specified periods, to mitigate inter- and intra-observer variability, as explained in a previous study [4].

Data on SMR (continuous variable) were collected specifically for this study, as previously discussed, and were not derived from the MDCS database.

### Statistics

The study population was at first divided into tertiles of low, moderate, and high SMR (low 1–1.26, moderate 1.26–1.41, high > 1.41) to identify potential associations with clinical and prognostic variables. For robustness, we wanted to compare large groups. Three groups were chosen instead of two to be able to identify a potential U-shaped association. Additionally, to be able to test our hypothesis that extensive spiculation might reflect a beneficial immune response and harbor prognostic significance, we conducted analyses comparing the 10% of women with the highest SMR values (*n* = 16) to the rest of the population (*n* = 145). For categorical tumor characteristics, the Chi-squared test and Fisher’s exact test were used to examine their relation to SMR. For continuous characteristics, ANOVA was employed, with log-transformed values applied where appropriate. Breast cancer-specific survival was analyzed using Kaplan–Meier curves, the log-rank test, and Cox regression analysis. The survival analyses were adjusted one by one for age at diagnosis (as a continuous variable), breast density (as an ordinal variable with three categories), and tumor size (as a continuous variable). In the Cox models, the proportional hazards assumption was tested using the method of Grambsch and Therneau [19], and no significant deviations were found. A *p*-value below 0.05 was considered statistically significant. No correction for multiplicity was applied, so *p*-values should be interpreted as descriptive statistics indicating potential associations rather than conclusive evidence. All analyses were performed using R version 4.3.0 [20].

## Results

From 2004 to December 31, 2014, a total of 680 women in the MDCS were diagnosed with breast cancer. Within this group, 259 had original radiology reports indicating a spiculated appearance. However, 98 women were excluded for various reasons: 16 lacked spiculated tumor appearance upon re-assessment, 76 had no available digital mammograms, three had bilateral breast cancer, and three had non-invasive breast cancer. Ultimately, 161 women, with a median age of 68 years (range 55–91) at the time of diagnosis, were eligible for inclusion in the study, Fig. 1.

Baseline characteristics for the full study population, as well as stratified by SMR tertiles, are presented in Table 1. Table 1 also includes *p*-values for associations between SMR and various patient and tumor characteristics. SMR was significantly associated with breast density (*p* = 0.030). High SMR was most common in women with fat-involuted breasts, 31% of whom had high SMR, whereas low SMR was most common in women with dense breasts, with 36% showing low SMR. The proportion of women who had a screening-detected breast cancer was highest in the women with a moderate SMR (78%). However, there were no significant differences regarding mode of cancer detection across SMR tertiles (*p* = 0.518), Table 1.

**Table 1:** Baseline population characteristics

Variable, *n* (%) if nothing else stated	All (*n* = 161)	Low (*n* = 56)	Moderate (*n* = 51)	High (*n* = 54)	*p*-value
Breast density					0.030
Fat involuted	36 (22)	10 (18)	9 (18)	17 (31)	
Moderately dense	86 (53)	26 (46)	29 (57)	31 (57)	
Dense	39 (24)	20 (36)	13 (25)	6 (11)	
Mode of cancer detection					0.518
Screening detected	118 (73)	41 (73)	40 (78)	37 (69)	
Clinically detected	43 (27)	15 (27)	11 (22)	17 (31)	
Age at diagnosis in years, median (range)	68 (55–91)	66 (56–87)	67 (55–85)	71 (55–91)	0.002
Tumor size in mm, median (range)	15 (5–50)	17 (5–50)	14 (7–50)	14 (5–33)	0.165^**^
Missing	3	0	0	3	
ER positivity	151 (96)	53 (95)	47 (96)	51 (98)	0.777^*^
Missing	4	0	2	2	
PR positivity	122 (79)	43 (77)	39 (80)	40 (80)	0.907
Missing	6	0	2	4	
HER2 receptor positivity	8 (5)	2 (4)	3 (6)	3 (6)	0.818^*^
Missing	6	2	1	3	
Ki67 expression					0.213
Low	54 (45)	21 (52)	18 (46)	15 (38)	
Intermediate	38 (32)	8 (20)	12 (31)	18 (45)	
High	27 (23)	11 (28)	9 (23)	7 (18)	
Missing	42	16	12	14	
Histological grade					0.051
I	49 (31)	11 (20)	22 (43)	16 (31)	
II	86 (54)	35 (62)	20 (39)	31 (60)	
III	24 (15)	10 (18)	9 (18)	5 (10)	
Missing	2	0	0	2	
ALNI	45 (28)	17 (30)	16 (31)	12 (24)	0.631
Missing	3	0	0	3	
Histological type					
Invasive ductal cancer	114 (73)	41 (73)	38 (76)	35 (69)	
Invasive lobular cancer	28 (18)	10 (18)	7 (14)	11 (22)	
Invasive tubular	10 (6)	2 (4)	5 (10)	3 (6)	
Other	5 (3)	3 (5)	0 (0)	2 (4)	
Missing	4	0	1	3	
Histological type binary: ductal YES or NO					0.708
Yes	114 (73)	41 (73)	38 (76)	35 (69)	
No	43 (27)	15 (27)	12 (24)	16 (31)	
Missing	4	0	1	3	

Baseline characteristics for the entire study population, and stratified into tertiles with low, moderate, and high SMR, respectively. Data presented as percentages unless otherwise stated

^*^ Fisher’s exact test

^**^ Analysis made on log-scale. Otherwise, a Chi-squared test is used for categorical variables. For continuous variables, an ANOVA test has been used. In the case of tumor size, the test was performed log of the values

There was also a significant positive association between age at diagnosis and SMR (*p* = 0.002), indicating that higher SMR values were more prevalent in older women.

**Fig. 3:**
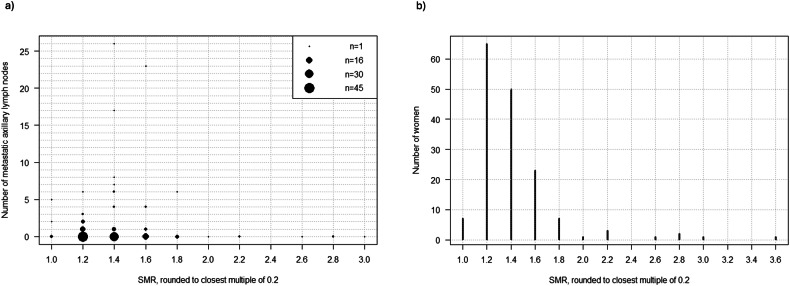
Visualizations of SMR values in the population and the relation to the number of metastatic lymph nodes in the axilla. **a** Number of metastatic axillary lymph nodes in relation to SMR values. The number of women with a given number of lymph nodes and a given SMR is proportional to the area of the circle. **b** Distribution of SMR values in the population, which illustrates the accumulation of SMR values between 1 and approximately 1.75

In contrast, ALNI was not significantly associated with SMR (*p* = 0.631). However, none of the women with an SMR ≥ 2 (*n* = 9) had ALNI, Fig. 3a. It is worth noting that the vast majority of women had SMR values below 2, Fig. 3b.

When comparing the 10% of women with the highest SMR values (SMR > 1.7, *n* = 16) to the remaining 90% of the population (*n* = 145), all women in the high-SMR subgroup had ER-positive tumors. Additionally, few had high Ki67 levels: among those with available Ki67 data, 92% had low or intermediate Ki67, compared to 75% in the rest of the population. Only one woman (7%) in the high-SMR subgroup had ALNI, compared to 44 women (31%) in the remaining group.

**Fig. 4:**
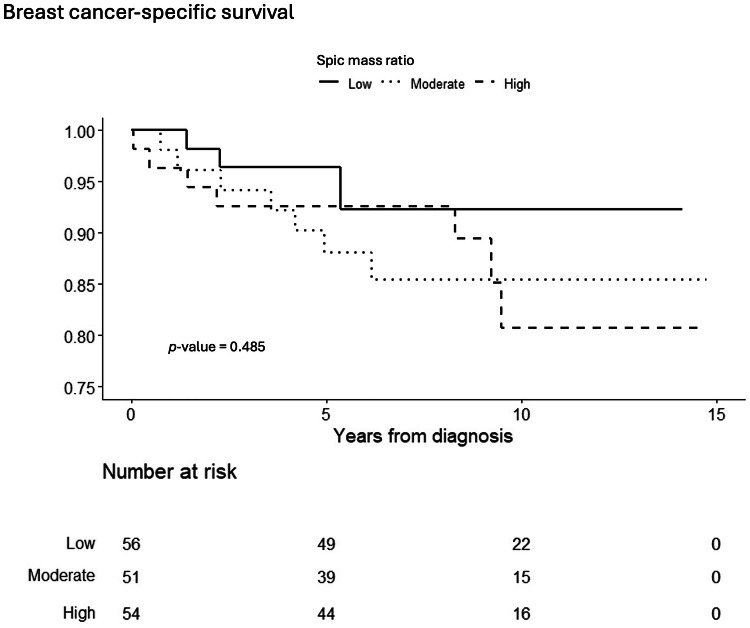
Kaplan–Meier graph illustrating breast cancer-specific survival for the population stratified by SMR (low 1–1.26, moderate 1.26–1.41, and high > 1.41). Overall *p*-value = 0.485

We observed no significant difference in breast cancer-specific survival between women with moderate or high SMR and those with low SMR, as depicted by the Kaplan-Meier curves (*p* = 0.485; Fig. 4). During the follow-up period, 18 women died from breast cancer, whereas 33 women died from other causes. In an unadjusted Cox regression, a non-significant increase in the hazard ratio (HR) for breast cancer death was identified for moderate and high SMR, as presented in panels *a* and *b* of Table 2. The *p*-value remained non-significant after adjusting for age, breast density, and tumor size. For clarity, Table 2 is divided into two panels, *a* and *b*, due to missing tumor size data for three individuals. Panel *a* displays the HR for breast cancer death, both unadjusted and adjusted for age at diagnosis and breast density, using the entire study cohort of 161 women. Panel *b* presents the survival analysis for the 158 women with available tumor size data. In panel *b*, the HRs for the subset with complete data (*n* = 158) resembled those for the whole cohort (*n* = 161) in panel *a*, showing a non-significant increase in HR for breast cancer death among women with moderate and high SMR compared to those with low SMR.

**Table 2:** Breast cancer-specific survival

a						
	Alive	Dead	Dead BC	HR_unadj_ (95% CI)	HR_adj_^*^ (95% CI)	HR_adj_^**^ (95% CI)
SMR						
Low	46	10	4	1	1	1
Intermediate	43	8	7	2.01 (0.59–6.89)	1.89 (0.55–6.47)	2.04 (0.59–6.99)
High	39	15	7	1.88 (0.55–6.42)	1.00 (0.28–3.54)	1.92 (0.55–6.65)
*p*-value (overall)				0.459	0.462	0.448
Observations	128	33	18			

b					
	Alive	Dead	Dead BC	HR_unadj_ (95% CI)	HR_adj_^***^ (95% CI)
SMR					
Low	46	10	4	1	1
Intermediate	43	8	7	2.03 (0.59–6.93)	1.78 (0.50–6.30)
High	38	13	5	1.36 (0.37–5.07)	1.80 (0.48–6.79)
*p*-value (overall)				0.509	0.578
Observations	127	31	16		

Cox regression analysis depicting breast cancer survival for women with different SMR levels. In other words, the hazard ratios (HR) apply to death in breast cancer (Dead BC). (**a**): adjusted for age at diagnosis, and breast density. (**b**): adjusted for tumor size (smaller sample due to missing data regarding tumor size for three individuals)

^*^ Adjusted for age at diagnosis only

^**^ Adjusted for breast density only

^***^ Adjusted for tumor size only

In summary, the analysis revealed two statistically significant findings: SMR was positively associated with age at diagnosis, indicating higher SMR with increasing age, and SMR was higher in fat-involuted breasts compared to moderately dense or dense breasts. No other significant associations were found between SMR and patient or tumor characteristics, including survival analysis.

## Discussion

In this study, we explored whether the SMR, which measures the degree of spiculation relative to tumor mass in spiculated breast cancer, is associated with tumor characteristics and breast cancer survival. Our findings indicate that SMR is higher in older women and those with fat-involuted breasts. However, we did not identify a statistically significant association between SMR and survival or an association with ALNI. Despite this, a potentially clinically important observation was that none of the women with an SMR ≥ 2 had metastatic ALNI. The small sample size (*n* = 9), however, limits the ability to draw definitive conclusions. Although spiculated breast cancers are generally linked to favorable tumor characteristics, our findings do not support the notion that the degree of spiculation, as measured by SMR in tertiles, is an independent prognostic factor. It is plausible that the visibility of spiculation is more strongly modulated by breast density and technical imaging parameters than by intrinsic tumor biology, which may attenuate any underlying prognostic associations.

To our knowledge, no prior research has specifically examined differences within spiculated breast tumors, despite studies linking spiculated tumors to clinical factors [21]. Several investigations have indicated that spiculated tumor appearances increase with age [6, 22, 23]. Ildefonso et al [6] reported that among the 234 spiculated tumors in their study, 76% were identified in women over 50, while 23% were found in women younger than 50. Similarly, Evans et al [23] found that the majority of women with spiculated masses in their cohort were over 60. These findings align with our results, suggesting that spiculation may develop over time, leading to more extensive spiculations in older individuals. Alternatively, the greater visibility of spiculations in older ages might be due to more prevalent fat-involved parenchyma [24]. Moreover, we observed that a high SMR frequently occurred in fat-involuted breasts, although results were not adjusted for age, suggesting that spiculations are more easily delineated in these breasts. A high body mass index (BMI) is linked with reduced breast density [25]; our recent publication demonstrated an association between spiculated masses and a BMI greater than 30 [26]. The positive associations between spiculation, age, and weight merit further investigation, primarily regarding breast density reduction with age. Understanding this relationship in a larger cohort would be beneficial, as the breast parenchyma’s microenvironment is thought to change with density, potentially affecting spiculation formation likelihood.

Moriuchi et al did not observe a difference in survival rates between women with spiculated breast cancer and those with non-spiculated tumors [22]. This finding aligns with our study on breast cancer survival within the MDCS, where all women with invasive breast cancer from the cohort (*n* = 1116) were included [27]. Conversely, some studies have reported improved survival for women with spiculated tumors compared to women with other tumor appearances [23, 28–30]. In our study, however, we did not identify significantly better survival in any subgroups when stratified by the degree of spiculation.

Active surveillance is increasingly being adopted for rectal, prostate, and urethral cancers [31–34]. In breast cancer, it is under investigation as a management strategy for low-grade ductal carcinoma in situ [35–38]. The ability to identify indolent cancer through imaging might also permit active surveillance for some patients with invasive breast cancer. Exploring the SMR further could be worthwhile. In our study, we used a basic measurement to assess the degree of spiculations. Implementing advanced techniques and automated quantifications, such as radiomics and artificial intelligence, could enhance prognostic predictions related to this phenomenon [39, 40] and is something we consider for future work. We further believe that spiculated lesions could be identified and classified more objectively with AI and radiomics, supporting and complementing radiologist evaluations. It could also improve the consistency in breast density assessment. Additionally, other imaging methods, including digital breast tomosynthesis and magnetic resonance imaging, could be considered for evaluating spiculations [41].

The primary limitation of this study is its small sample size, which includes a limited number of events in the survival analysis. Long follow-up time is crucial when studying breast cancer survival. A surrogate measure that can be used instead of survival is ALNI, which we have also studied in addition to survival. Although SMR is defined, no explicit operational boundary was used to separate the tumor core from the spiculations; this may attenuate associations and limit generalizability. It is also worth noting that an inter-reader agreement for SMR could not be estimated. This could have introduced an unquantified measurement error, which reinforces the exploratory nature of the findings. Additionally, stratifying the SMR into tertiles (low, moderate, and high) may be too crude to effectively assess potential associations. Case identification in this study was based on radiological reports describing spiculation (rather than an independent mammography re-read of all breast cancers diagnosed within the study time frame). This mode of identification can have introduced a small, non-quantifiable selection bias. Breast density was qualitatively assessed, reflecting clinical practice. Although qualitative assessment may introduce misclassification and interfere with associations, it enhances applicability. No formal assessment of intra- or inter-observer variability was conducted, but a previous study using the same radiologists and density classification system showed substantial inter-observer agreement [42]. Missing data proportion for Ki67 reflects the retrospective design; however, prior analyses suggest data are missing at random, supporting valid internal comparisons [4]. We acknowledge the risk of multiple testing in this setting, which also has power limitations, but in this exploratory study with a small sample size, we prioritized identifying potential signals for future research.

## Conclusions

The SMR, which measures the degree of spiculations relative to tumor mass, was not significantly associated with breast cancer survival or ALNI. Notably, none of the cases exhibiting a very high ratio (≥ 2) showed ALNI. However, the sample size was limited, and these results should be seen as exploratory and of a descriptive nature. Further research on a larger scale is necessary to explore the prognostic implications of extensive spiculation in spiculated breast cancer.

## Data Availability

The dataset from the Malmö Cohorts supporting the conclusions of this article was used under a license and is not available as open source. Please visit their website for more information (https://www.malmo-kohorter.lu.se/malmo-cohorts).

## Abbreviations

ALNI: Axillary lymph node involvement
BI-RADS: Breast imaging reporting and data system
BMI: Body mass index
ER: Estrogen receptor
HER2: Human epidermal growth factor receptor 2
MDCS: Malmö diet and cancer study
PR: Progesterone receptor
SMR: Spic mass ratio
